# High pulse pressure is a risk factor for prodromal Alzheimer’s disease: a longitudinal study

**DOI:** 10.18632/aging.103678

**Published:** 2020-09-22

**Authors:** Wen-Yan Shi, Zuo-Teng Wang, Fu-Rong Sun, Ya-Hui Ma, Wei Xu, Xue-Ning Shen, Qiang Dong, Lan Tan, Jin-Tai Yu, Yang Yu, Alzheimer’s Disease Neuroimaging Initiative

**Affiliations:** 1 Department of Neurology, Qingdao Municipal Hospital, Dalian Medical University, Dalian, China; 2 College of Medicine and Pharmaceutics, Ocean University of China, Qingdao, China; 3 Department of Neurology, Qingdao Municipal Hospital, Qingdao University, Qingdao, China; 4 Department of Neurology and Institute of Neurology, Huashan Hospital, Shanghai Medical College, Fudan University, Shanghai, China; 5 Department of Neurology, The Affiliated Hospital of Qingdao University, Qingdao, China

**Keywords:** pulse pressure, Alzheimer’s disease, risk, non-demented

## Abstract

It has been increasingly evident that pulse pressure (PP) is associated with Alzheimer's disease (AD) but whether PP increases AD risk and the mechanism responsible for this association remains unclear. To investigate the effects of PP in the process of AD, we have evaluated the cross-sectional and longitudinal associations of PP with AD biomarkers, brain structure and cognition and have assessed the effect of PP on AD risk in a large sample (n= 1,375) from the Alzheimer’s Disease Neuroimaging Initiative (ADNI). Multiple linear regression and mixed-model regression were employed in cross-sectional and longitudinal analyses respectively. Clinical disease progression was assessed using Cox proportional hazards models. High PP was associated with lower β-amyloid 42 (Aβ_42_) (*P*= .015), and higher total tau (T-tau) (*P*= .011), phosphorylated tau (P-tau) (*P*= .003), T-tau/Aβ_42_ (*P*= .004) and P-tau/Aβ_42_ (*P* = .001), as well as heavier cortical amyloid-beta burden (*P*= .011). Longitudinally, baseline high PP was significantly associated with hippocampal atrophy (*P*= .039), entorhinal atrophy (*P*= .031) and worse memory performance (*P*= .058). Baseline high PP showed more rapid progression than those with normal PP (*P* <.001). These results suggest PP elevation could increase AD risk, which may be driven by amyloid plaques and subclinical neurodegeneration.

## INTRODUCTION

Alzheimer’s disease (AD) is a neurodegenerative disorder of elderly individuals and is characterized by the accumulation of β-amyloid and tau in brain, progressive brain atrophy, and cognitive decline [1]. With the increase of life expectancy in developed countries, the incidence of AD and its socioeconomic impact are also growing [2]. Currently, there is no preventive or disease-modifying therapeutic measures, therefore identification of modifiable risk factors is required. The well-established AD biomarkers include cerebrospinal fluid (CSF) biomarkers of β-amyloid 42 (Aβ_42_), total tau (T-tau), and phosphorylated tau (P-tau); positron emission tomography (PET) measurements of Aβ and tau; and structural magnetic resonance imaging (MRI) measurements [3–5], which are increasingly used to support the diagnosis of AD in research, clinical practice and drug development and have become part of the newly revised early diagnostic criteria for AD [6, 7]. Therefore, it is necessary to investigate the associations between susceptibility factors and biomarkers in the preclinical stage of AD, which is helpful for early identification of modifiable factors.

Pulse pressure (PP) is an index of vascular aging and displays a linear increase with age [8]. It has also been recognized as a marker of increased arterial stiffness and widespread atherosclerosis. Atherosclerosis and cerebrovascular diseases have been implicated in the occurrence and development of AD [9, 10]. Therefore, it is biologically plausible to suppose that high pulse pressure could be related to the development of AD [11]. Several relevant studies indicated that high PP was associated with AD pathophysiology [8, 12–14], suggesting that vascular aging might increase AD risk [11]. Some studies suggested PP elevation was associated with CSF P-tau and Aβ_42_ in cognitively normal older adults [8]. Others found arterial stiffness was associated with Aβ plaque deposition in the brain [15]. Furthermore, previous studies indicated arterial stiffness might play a role in early cognitive decline and brain atrophy in mid-to-late life [13, 14, 16]. However, these studies were mostly limited by their cross-sectional design and small samples, and the mechanisms underlying the association between PP and AD were still unclear.

More studies are warranted to explore whether PP might increase AD risk or not. This study was designed to investigate whether PP was related to baseline and longitudinal changes in AD biomarkers such as CSF biomarkers, cortical amyloid-beta load, MRI measurements and neuropsychological composites in a large sample of non-demented elderly from the Alzheimer’s Disease Neuroimaging Initiative (ADNI) study.

## RESULTS

### Demographic and clinical data

There were 669 people with high PP and 706 with normal PP in the ADNI. Comparisons of characteristics between groups were presented in Table 1. Participants in the high PP group were more likely to be older (*P* < .001) and hypertension (*P* < .001) than those with normal PP. But there was no group difference in gender, education, *APOE* Ɛ4 carrier status, and other vascular risk factors (all *P* > .050). Clinical profiles of patients changed drastically due to data availability issues, the new selected sub-datasets were presented in Supplementary Table 1.

**Table 1:** Participant demographic and clinical information.

**Participant features**	**Normal PP (<60mmHg)**	**High PP (≥60mmHg)**	***P* Value**
**N**	706	669	
**Age(Mean ± SD, year)**	72.31±6.91	74.73±6.86	<0.001
**Gender (M/F)**	396/310	366/303	0.606
**Education (Mean ± SD, year)**	16.19±2.76	16±2.83	0.214
***APOE* Ɛ4 carrier status (2/1/0)**	59/248/399	47/226/396	0.500
**BMI (Mean ± SD, kg/m^2^)**	27.1±4.84	26.94±4.78	0.373
**CVD(yes/no)**	156/550	154/515	0.682
**Hyperlipemia (yes/no)**	329/377	320/349	0.647
**Hypertension (yes/no)**	296/410	347/322	**<0.001**
**T2DM (yes/no)**	44/662	60/609	0.055
**Cognitive diagnoses**			
**CN/MCI**	255/451	259/410	0.320

Abbreviations: Normal PP=normal pulse pressure; High PP=high pulse pressure; SD=standard deviation; *APOE*, *apolipoprotein epsilon*; BMI, Body Mass Index; CVD, Cardiovascular Disease; T2DM, Type 2 diabetes mellitus; CN, cognitively normal; MCI, mild cognitive impairment.

### Pulse pressure and CSF biomarkers

**Figure 1:**
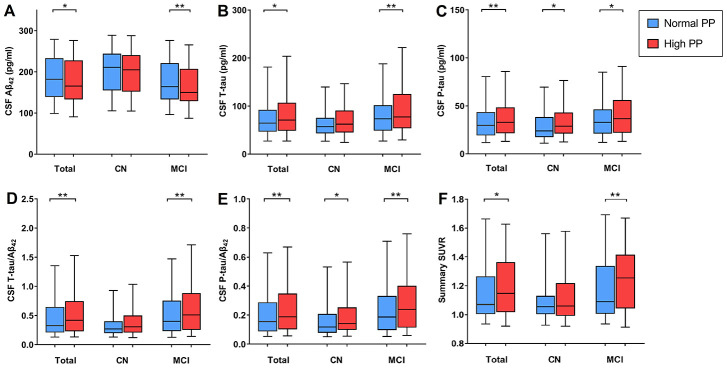
**Association between pulse pressure (PP) and AD biomarkers at baseline.** (**A**) PP is negatively correlated with CSF Aβ_42_ within non-dementia and MCI groups; (**B**) PP is positively correlated with CSF T-tau within non-dementia and MCI groups; (**C**) PP is positively correlated with P-tau in all diagnostic groups; (**D**) PP is positively correlated with CSF T-tau/ Aβ_42_ within non-dementia and MCI groups; (**E**) PP is positively correlated with CSF P-tau/Aβ_42_ in all diagnostic groups; (**F**) PP was positively correlated with cortical Aβ load in summary SUVR within non-dementia and MCI groups. Abbreviations: AD, Alzheimer’s disease; CSF, cerebrospinal fluid; CN, cognitively normal; MCI, mild cognitive impairment; Aβ, β-amyloid; SUVR, standardized uptake value ratio. *p<.05; **p<.01; *** *p*<.001.

CSF measurements were available for 977 non-demented participants at baseline (n = 364 CN, 613 MCI), of whom 517 had normal PP. In cross-sectional analyses, after excluding extreme outlines, high PP was associated with a decrease in Aβ_42_ (β = -.525, *P* = .015, Figure 1A), as well as increases in T-tau (β = .077, *P* = .011, Figure 1B), P-tau (β = .097, *P* = .003, Figure 1C), T-tau/Aβ_42_ (β = .131, *P* = .004, Figure 1D) and P-tau/Aβ_42_ (β = .157, *P* = .001, Figure 1E) after adjustment for age, gender, education, *APOE* Ɛ4 carrier status, vascular risk factors, cognitive diagnosis and extracted CSF volume. When stratified by cognitive diagnosis, the associations between PP and CSF biomarkers still persisted within MCI group and high PP was associated with higher P-tau and P-tau/Aβ_42_ levels in CN group (Figure 1C and 1E); when stratified by age, the associations between PP and CSF biomarkers still persisted within very old group and PP elevation was associated with increased T-tau/Aβ_42_ in young old group (Supplementary Table 2).

There were 526 people who had at least one follow-up visit at baseline enrolled in the five-year longitudinal analysis. Longitudinally, we did not find any association between baseline PP and CSF biomarkers. Similarly, no associations were detected when the analyses were stratified by cognitive diagnosis (Supplementary Table 2), while baseline high PP was associated with lower T-tau/Aβ_42_ in the young old subgroup when stratified by age (Supplementary Table 2).

### Pulse pressure and AV45 PET imaging

The mean Aβ load measured by the florbetapir AV45 standardized uptake value ratio (SUVR) was available in 739 participants at baseline (n = 280 CN, 459 MCI), of whom 358 had high PP. In cross-sectional analyses, after excluding extreme outlines, we found that PP was positively correlated with cortical Aβ load in summary SUVR (β = .018, *P*=.011, Figure 1F) when adjusted for age, gender, education, *APOE* Ɛ4 carrier status, vascular risk factors, cognitive diagnosis and florbetapir mean of composite ref region. When stratified by cognitive diagnosis and age, the association between PP and cortical Aβ load remained significant within MCI (β = .034, *P* = .003, Figure 1F) and the young old group (Supplementary Table 2).

There were 550 people who had at least one follow-up visit at baseline enrolled in the five-year longitudinal analysis. Longitudinally, we did not identify a statistically significant association between PP and summary SUVR (β = 1.457*e^-3^, *P* = .144). Similarly, no associations were detected when the analyses were stratified by cognitive diagnosis or age (Supplementary Table 2).

### Pulse pressure and MRI measurements

Measurements of hippocampal, entorhinal, and mid-temporal volumes were available in 1,137 participants at baseline (n = 694 MCI), of whom 554 had high PP. In cross-sectional analyses, after excluding extreme outlines, increased PP was not associated with hippocampal volume (β = -1.084*e^3^, *P* = .312), entorhinal volume (β = -2.311*e^1^, *P* = .554), or mid-temporal volume (β = 4.364*e^1^, *P* = .752) when adjusted for age, gender, education, *APOE* Ɛ4 carrier status, vascular risk factors, cognitive diagnosis and intracranial volume. Similarly, no associations were detected between PP and MRI structure when the analyses were stratified by cognitive diagnosis and age (Supplementary Table 3).

**Figure 2:**
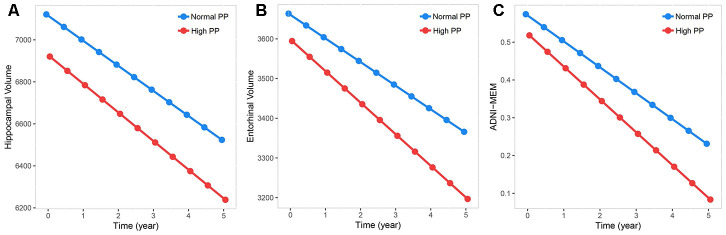
**Associations between baseline pulse pressure and measurements of brain aging.** Data from linear mixed-effects models adjusted for age, gender, education, *APOE* Ɛ4 carrier status, vascular risk factors, cognitive diagnosis, as well as intracranial volume. Increased PP level was associated with an accelerated decline in measurements of brain aging. (**A**–**C**) Increased PP level was associated with accelerated decline in hippocampal volume, entorhinal volume and episodic memory performance.

There were 1,042 who had at least one follow-up visit at baseline enrolled in the five-year longitudinal analysis. Longitudinally, increased PP was associated with an accelerated decline in hippocampal volume (β = -16.903, *P* = .039, Figure 2A) and entorhinal volume (β = -20.014, *P* = .031, Figure 2B) over time. When stratified by cognitive diagnosis and age, the association between PP and entorhinal volume still persisted among those with MCI and the young old subgroup (Supplementary Figure 1A and Table 3).

### Pulse pressure and ADNI-MEM & ADNI-EF

ADNI-MEM and ADNI-EF were available in 1,375 (n = 514 CN, 861 MCI) participants at baseline, of whom 706 had normal PP. In cross-sectional analyses, no association of PP was found with memory performance (β = −.030, *P* = .343) or executive function performance (β = −.042, *P* = .298). Similarly, no association was detected when the analyses were stratified by cognitive diagnosis while high PP was associated with worse memory performance in young old group when stratified by age (Supplementary Table 3).

There were 1322 who had at least one follow-up visit at baseline enrolled in the five-year longitudinal analysis. Longitudinally, increased PP was associated with worse memory performance with a strong tendency towards statistical significance (β = -1.829*e^-2^, *P* = .058, Figure 2C). In stratified analyses restricted to MCI participants, increased PP was associated with a greater decline in memory performance over time (β = −.034, *P* = .012, Supplementary Figure 1B and Table 3). We did not find any statistically significant associations in other stratified analyses (Supplementary Table 3).

### Pulse pressure and clinical disease progression

**Figure 3:**
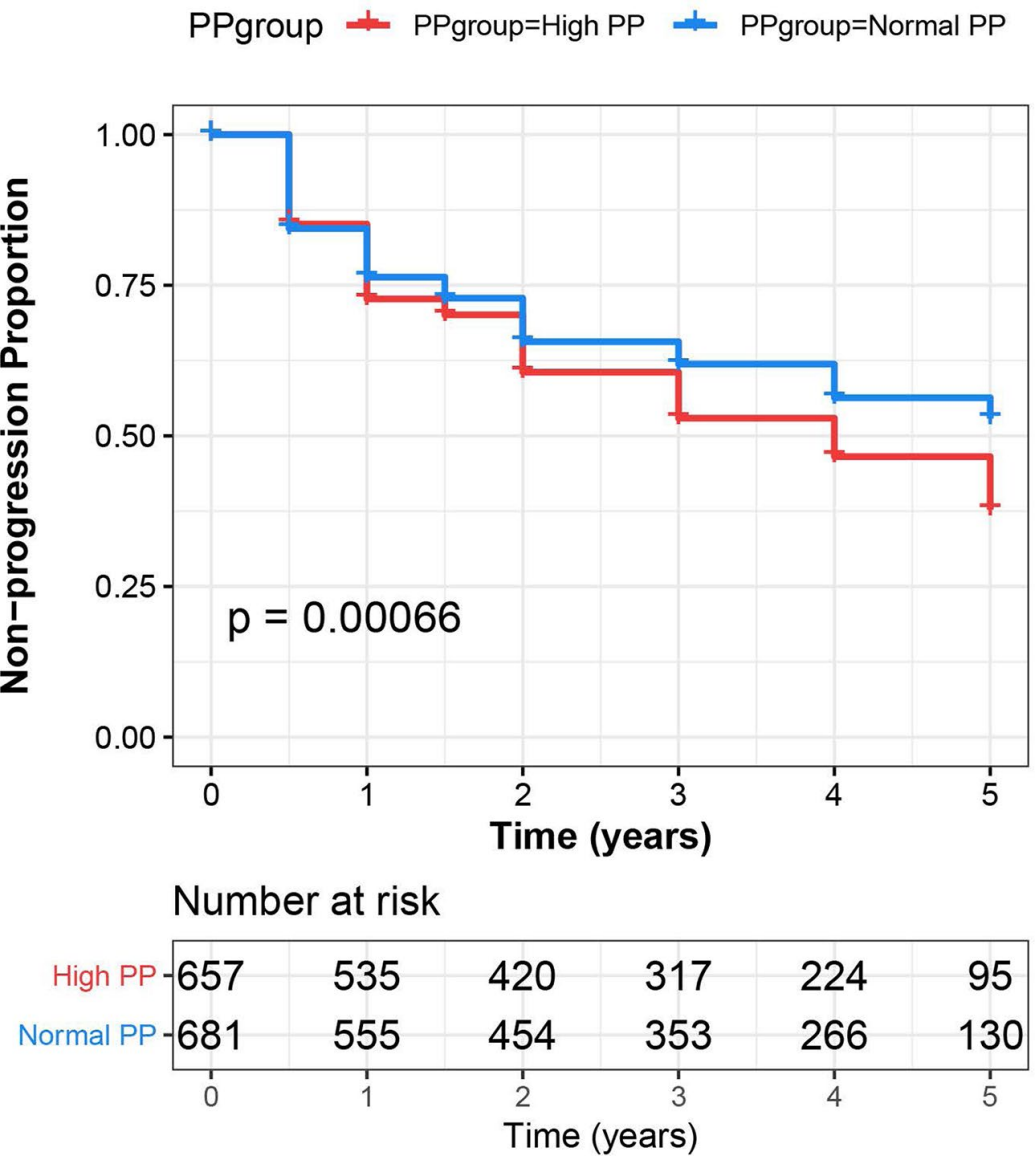
**Pulse pressure predicts more rapid progression to dementia.** The Cox regression indicates that a higher pulse pressure was associated with more rapid progression to dementia. Visually, the survival plot displays results for high pulse pressure in comparison with normal pulse pressure.

Kaplan-Meier analysis revealed participants with high PP at baseline showed more rapid progression over the following five years, compared with those with normal PP (*P* <.001, Figure 3). In Cox regression models (adjusted for age, gender, education, *APOE* Ɛ4 carrier status, vascular risk factors and cognitive diagnosis), the individuals with high PP had a higher risk of progression to AD (hazard ratio 1.216, 95% CI 1.051-1.461, *P* = .011).

### Systolic blood pressure (SBP), diastolic blood pressure (DBP) and hypertension

SBP exhibited the same pattern of associations with CSF biomarkers found in the PP analyses. Higher SBP was associated with a decrease in Aβ_42_, as well as increases in T-tau, P-tau, T-tau/Aβ_42_ and P-tau/Aβ_42_ (Supplementary Table 4); in AV45 PET imaging analyses, SBP was positively correlated with cortical Aβ load in summary SUVR, and the association still persisted within MCI and the young old subgroup (Supplementary Table 4); in MRI measurements analyses, we only found that higher SBP was associated with smaller mid-temporal volume within MCI (Supplementary Table 4); in ADNI-MEM and ADNI-EF analyses, higher SBP was associated with worse memory performance and executive function performance in the young old subgroup (Supplementary Table 4).

There was no association between DBP and CSF biomarkers or AV45 PET imaging analyses (Supplementary Table 5); in MRI measurements analyses, DBP was negatively correlated with mid-temporal volume and it still persisted within MCI subgroup (Supplementary Table 5); in ADNI-MEM and ADNI-EF analyses, higher DBP was associated with worse memory performance, and when stratified by age, the association still persisted within very old subgroup while higher DBP was associated with worse executive function performance in MCI subgroup when stratified by cognitive diagnosis (Supplementary Table 5).

Hypertension exhibited the same pattern of associations with CSF biomarkers and AV45 PET imaging found in the DBP analyses (Supplementary Table 6); in MRI measurements analyses, we found hypertension was negatively correlated with hippocampal volume, entorhinal volume, and mid-temporal volume in the young old subgroup (Supplementary Table 6); in ADNI-MEM and ADNI-EF analyses, hypertension was associated with worse executive function performance, and when stratified by age and cognitive diagnosis, the association still persisted within MCI, the young old and very old subgroups while association between hypertension and memory performance was found in the young old subgroup (Supplementary Table 6).

## DISCUSSION

In this study, PP elevation was found to be associated with CSF Aβ_42_, T-tau, P-tau, T-tau/ Aβ_42_ and P-tau/Aβ_42_, as well as cortical Aβ load at baseline; and longitudinally, an increase in PP was associated with an accelerated decline in hippocampal and entorhinal volumes, and with worsening episodic memory. These associations seemed to be more obvious in MCI and very old patients, suggesting that the relationships between pulse pressure and cognitive disorders were age- and diagnosis- dependent [17]. Individuals with higher PP also had a higher incidence of conversion to AD. Taken together, these findings supported that PP elevation could increase risk of AD, and the associations maybe driven by amyloid plaques and subclinical neurodegeneration, which was consistent with the conclusion from previous studies that elevated PP had a negative impact on hallmark neuropathological markers of AD [8, 12, 13, 18]. These likely suggested PP can be added to the current dementia risk models for dementia prevention, if controlled effectively, it would help delay the onset and reduce the number of demented people in the future.

In CSF biomarkers analyses, when stratified by cognitive diagnosis, the associations between PP and P-tau, P-tau/Aβ_42_ still persisted among MCI and CN groups, while the relationship between Aβ_42_ and PP only persisted within MCI group, which possibly revealed that the relationship between PP and P-tau was detected at early stages. These findings indicated that PP may be related to both amyloid plaques and tau-mediated neurodegeneration, and the latter mechanism may be more salient, which was consistent with previous articles [8, 19]. Although AD is characterized by both amyloid- and tau-based pathologies, P-tau is more strongly associated with neurodegeneration and cognitive decline; besides, we also find that PP is associated with brain atrophy and cognitive decline, suggesting that high PP may convey AD risk through its closer association with tau phosphorylation [8]. Previous work has indicated that amyloid is not cause of AD but the downstream result [20] and nearly 23% of elderly exhibit P-tau elevation in the absence of amyloidosis [21]. And the update of an AD model indicates that neurodegeneration may occur independently and ahead of amyloid pathology and may be exacerbated by the later development of amyloidosis [22, 23]. However, the underlying pathological mechanisms warrant further investigation. Longitudinally, baseline high PP was associated with lower T-tau/Aβ_42_ in the young old subgroup, it seemed conflicting, which may be explained by the large number of subjects lost to follow-up, especially in the 3-, 4-, 5-year follow-up.

In MRI analyses, increased PP accelerated the decrease in hippocampal and entorhinal volumes, which was consistent with the previous finding that blood pressure can preferentially affect the hippocampal volume [24]. Though the responsible mechanism linking PP to reduced hippocampal volume has not been elucidated, Beauchet et.al have showed preferential global and regional effects of blood pressure on the brain, including the hippocampus [25]. These effects may be mediated in part by blood pressure-related arteriolosclerosis, low blood flow, and consequent hypoperfusion in the hippocampal [25]. Importantly, loss of vascular elasticity and increased vascular resistance, caused partly by increased vascular amyloid deposit, may mediate such effects [26–28].

Despite the growing recognition that vascular risk factors may have an impact on the development of AD, the pathophysiological mechanism needs further understanding. In fact, mounting evidence suggests that the pulsation of the arteries contributes to the clearance of wastes from the central nervous system [29, 30]. Besides, some studies suggest that circulatory injuries, such as those caused by stiffening of the vasculature system, may result in failure of clearance of Aβ from the brain [31]. To be more specific, elevated PP may stimulate vascular hypertrophy, remodeling, or rare in the microcirculation, leading to increased vascular resistance, impaired microvascular reserve [32–34], which may subsequently cause structural changes, impair clearance of Aβ_42_ along the perivascular spaces [35], as well as decrease arterial pulsatility and capacity for amyloid drainage [36]. Therefore, dysfunction of this system may promote neurodegeneration [37]. It is also possible that changes in vascular function could lead to reduced tissue perfusion and arteriolar hypercontractility, or blood-brain barrier (BBB) leakage, either of which may result in neurodegeneration and increased P-tau [38]. More animal-model studies are needed to shed light on the potential mechanisms via which PP influences AD.

Although PP was the primary focus of the study, we also examined SBP, DBP and hypertension in relation to biomarkers to determine their contributions to PP. We found that increased SBP was associated with CSF biomarkers, cortical Aβ load, brain volume and cognition, and the associations seemed to be more obvious in MCI and very old patients, which revealed that SBP may exhibit the similar pattern of association found in PP, while associations between DBP, hypertension and AD biomarkers mainly reflected in MRI measurements and cognition, this likely reflected the greater relative contributions of SBP to PP. PP elevation represented either increased SBP or decreased DBP, which may provide insight into the relationship between blood pressure and neurodegeneration. Furthermore, the associations above supported that higher SBP, DBP and hypertension may increase AD risk.

In Table 1, we found participants in the high PP group were more likely to be T2DM (*P* = .055), it seemed a link between PP and T2DM in AD, which may be mediated in part by vascular injuries. Some studies suggest that micro-vascular damage, sympathetic damage, and enhanced renin-angiotensin system, caused by diabetes mellitus, may aggravate systolic blood pressure elevation [39], resulting in high PP; besides, vascular damage such as arteriosclerosis caused by T2DM glycosylation was associated with low blood flow, leading to BBB leakage [40], which may result in neurodegeneration.

The study have several limitations. (1) Although the relationship between PP and dementia is supported by longitudinal analyses in the present study, the attrition bias due to loss to follow-up was not corrected in the analyses. Future studies with larger sample sizes, longer follow-up duration, and lower attrition rates will assist in exploring whether the associations support causality; (2) blood pressure was not an a priori outcome in the ADNI study and its assessment did not employ strict standards (such as average of multiple measurements), which may result in measurement bias; (3) the sample of participants who received pressure-controlled treatment (like anti-hypertension medicine) was small, therefore the analyses about it didn’t performed.

In conclusion, this large-scale study identified the cross-sectional as well as the longitudinal associations of PP and the known biomarkers of AD, suggesting high PP could increase AD risk, and found the PP effects may be modified by cognitive diagnosis and age, and may be driven by amyloid plaques and subclinical neurodegeneration. Furthermore, our study encourages future studies to consider PP as a target for AD prevention. However, the potential pathological mechanisms linking age-related vascular stiffening to neurodegeneration warrant further investigation, such as reduced brain blood flow, increased blood-brain barrier permeability, and decreased clearance of misfolded proteins, and pharmacological modulation in human subjects or configured animal models maybe helpful to elucidate the underlying mechanisms.

## MATERIALS AND METHODS

### ADNI

The data used for this analysis were downloaded from the ADNI database (adni.loni.usc.edu). The ADNI, an ongoing, multisite longitudinal, large-scale study launched in 2003, was designed to develop clinical, imaging, genetic and biochemical biomarkers for the early detection and tracking of AD [41]. Participants in the ADNI study underwent baseline and periodic physical and neurological examinations, standardized neuropsychological assessments, and biological sampling (blood, urine, and CSF) [13]. Regional ethical committees of all participating institutions approved the ADNI. All study participants provided written informed consent.

### Participants

**Figure 4:**
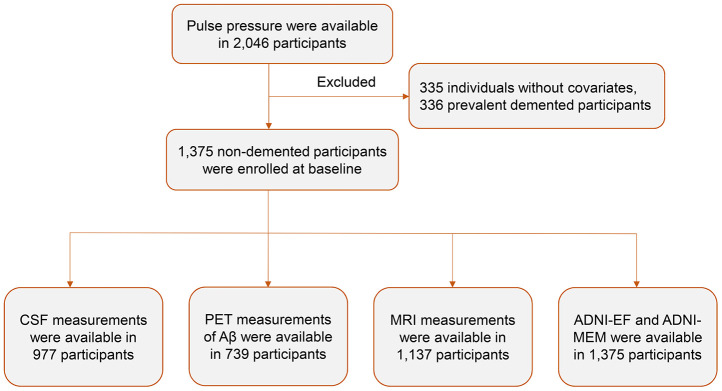
**Flow diagram of participant selection.** Abbreviations: CSF, cerebrospinal fluid; MRI, magnetic resonance imaging.

A total of 2,046 participants from ADNI1, ADNI Grand Opportunity, and ADNI 2 completed the blood pressure assessment at baseline. Among them, 335 participants without demographic and clinical information, and 336 who were classified as dementia were excluded. Finally, the remaining 1,375 non-demented participants were enrolled in this study (Figure 4). Clinical disease progression was ascertained for a large subset of participants (n = 1,338) who were followed up with serial clinical assessments at varying intervals for different length of time ranging from 0 year to 5 years.

### PP measurements

Seated brachial artery SBP and DBP were obtained and PP was calculated as the difference between SBP and DBP. PP of 60 mmHg or higher was defined as high PP [42].

### Covariates

Accumulating evidence supports a role of vascular risk factors in the development and etiology of AD [43]. For purposes of this study, participant medical history data of vascular risk factors was obtained until the date of baseline blood pressure. The covariates consisted of age, gender, education, *APOE* Ɛ4 carrier status, body mass index (BMI) which was calculated as weight in kilograms divided by the square of height in meters, and vascular risk factors, such as type 2 diabetes mellitus (T2DM), hyperlipemia, hypertension, as well as medical history of cardiovascular disease (CVD) including myocardial infarction, angina, heart failure and atrial fibrillation. We classified these diseases based on the medical history information and/or use of anti-medications.

### CSF biomarker measurements

The CSF collection and procedural protocols have been described previously [44]. All participants underwent lumbar puncture which was performed with a 20- or 24-gauge spinal needle as described in the ADNI procedures manual (http://www.adni-info.org/) and AD biomarkers including Aβ_1-42_, P-tau, and T-tau were measured using the multiplex xMAP Luminex platform (Luminex Corp, Austin, TX) with Innogenetics (INNO-BIA AlzBio3; Ghent, Belgium; for research use–only reagents) immunoassay kit–based reagents. All tests were administered at baseline and at 12, 24, 36, 48 and 60 months.

### 18F florbetapir AV45 PET imaging

Preprocessed florbetapir imaging data were downloaded from the LONI ADNI site (http://adni.loni.usc.edu). The data preprocessing is accessible online (adni. loni. ucla.edu/about-data-samples/image-data/). For quantifying cerebral cortical Aβ, preprocessed florbetapir image data and co-registered structural MRI were analyzed using Freesurfer (version 4.5.0) (surfer.nmr. mgh.harvard.edu/) as described previously and online (adni.loni.ucla.edu/research/pet-post-processing/). Briefly, image data were acquired in four 5-min frames 50–70 minutes after injection of approximately 10mCi of 18F florbetapir, the four frames were co-registered to one another, averaged, interpolated to a uniform image and voxel size (160×106×96, 1.5 mm^3^), and smoothed to a uniform resolution (8 mm FWHM) to account for differences between scanners [45]. The mean Aβ retention, measured by the florbetapir AV45 SUVR, was normalized to the whole cerebellum as a summary measure of florbetapir retention for each participant in cross-sectional analyses; and a composite reference region, which was made up of whole cerebellum, brainstem/pons, and eroded subcortical white matter, has been evaluated for longitudinal analyses. All tests were administered at baseline and at 12, 24, 36, 48 and 60 months.

### Brain structure

The process for MRI acquisition has been described elsewhere in ADNI publications [2, 46–48]. Structural brain images were acquired using 1.5T or 3T MRI systems with T1-weighted scans using a sagittal volumetric magnetization-prepared rapid acquisition gradient echo sequence. The ADNI project offers scans that have been preprocessed (gradient warping, scaling, B1 correction, and N3 inhomogeneity correction) to correct for different scanners across sites [49]. All tests were administered at baseline and at 3, 6, 12, 18, 24, 36, 48 and 60 months.

### Neuropsychological composites

The ADNI neuropsychological protocol, including calculation of ADNI-MEM and ADNI-EF composite measures, has been reported previously [50, 51]. The ADNI-MEM included a composite *z* score based on item-level data from the Rey Auditory Verbal Learning Test, the Mini-Mental State Examination (MMSE), the AD Assessment Scale Cognitive Test, and Logical Memory I and II. The ADNI-EF included item-level data from the Trail Making Test Parts A and B, Digit Span Backward, Digit Symbol, Animal Fluency, Vegetable Fluency, and Clock Drawing Test [52]. All tests were administered at baseline and at 6, 12, 18, 24, 36, 48 and 60 months.

### Clinical disease progression

CN and MCI participants were divided into group of clinical disease progression and stable, respectively. Participants were defined as having clinical disease progression if their global CDR/MMSE or clinical classification score changed (CN subjects converted to MCI or AD, or their global CDR scores rose to 0.5 or greater; MCI subjects lost more than 3 points between first and last MMSE, or converted to AD at follow-up, or got a score less than 24 on the last MMSE) [53–55]. If the above criteria have not been met at follow-up, participants were considered stable; regardless of the lost of subjects, once the progression criteria have been met during 5-year follow-up, they were deemed progressive.

### Statistical analyses

Baseline demographic, clinical and diagnostic characteristics were compared between PP groups using Mann-Whitney U test for continuous variables and χ2 analyses for categorical variables, respectively. We used means and standard errors for continuous measures and proportions for categories. Multiple linear regression was used to explore the association between PP and biomarkers in cross-sectional analyses after adjusting for age, gender, education, *APOE* Ɛ4 carrier status, vascular risk factors and cognitive diagnosis at baseline. Before regression analyses, participants who had a value >3 or <3 SD from the mean value were regarded as extreme outlines and excluded. In case of skewed distribution (Shapiro-Wilk test > 0.05) of biomarker data, transformation was performed to approximate a normal distribution via “car” package of R software. Interaction terms for age were used to explore whether strata effect existed, in order to minimize the difference between subgroup sample sizes, we chose 75 years old as the cutoff value (<75 years old vs. ≥ 75 years old). In case of any potential interactions (*P* < 0.1), subgroup analyses were further performed. Mixed-model regression with time modeled as years from baseline for each participant was used to explore the longitudinal influences of PP at baseline on AD biomarkers and cognition after adjusting with age, gender, education, *APOE* Ɛ4 carrier status, vascular risk factors, cognitive diagnosis. The time-by-exposure interaction terms tested whether PP were associated with changes in the given outcomes (CSF biomarkers, AV45 PET imaging biomarkers, MRI structure, as well as ADNI-MEM and ADNI-EF) over the follow-up period. Kaplan-Meier survival analysis investigated the relationship between baseline PP and clinical disease progression using years to cognitive decline as the time variable. Cox proportional hazards models (adjusted for age, gender, education, *APOE* Ɛ4 carrier status, diagnosis, and vascular risk factors) were used to test the predictive ability of baseline PP for clinical disease progression. All tests were two-tailed. Statistical significance was set at *P* < .05. R version 3.5.1 and GraphPad Prism 7.00 software were used for statistical analyses and figure preparation.

Although PP was the focus of the study, primary cross-sectional analyses which were identical to those used in PP were repeated to examine SBP (< 120mmHg, ≥ 120 and < 140mmHg, ≥ 140mmHg), DBP (< 80mmHg, ≥ 80 and < 90mmHg, ≥ 90mmHg) [56], and hypertension (based on the medical history information and/or use of anti-medications) in relation to biomarkers to determine their contributions to the PP findings. We did this because it was highly correlated with SBP and disambiguated the relative contributions of systolic and diastolic pressure to results, which may have provided mechanistic insight.

## Supplementary Material

Supplementary Figure 1

Supplementary Tables
