# Supplementary material for: High pulse pressure is a risk factor for prodromal Alzheimer’s disease: a longitudinal study
Source: Aging (Albany NY). 2020 Sep 22;12(18):18221–37. doi: 10.18632/aging.103678 (PMC7585106; doi:10.18632/aging.103678)
Supplement: Supplementary Figure 1 [file aging-12-103678-s001..pdf]

SUPPLEMENTARY FIGURE

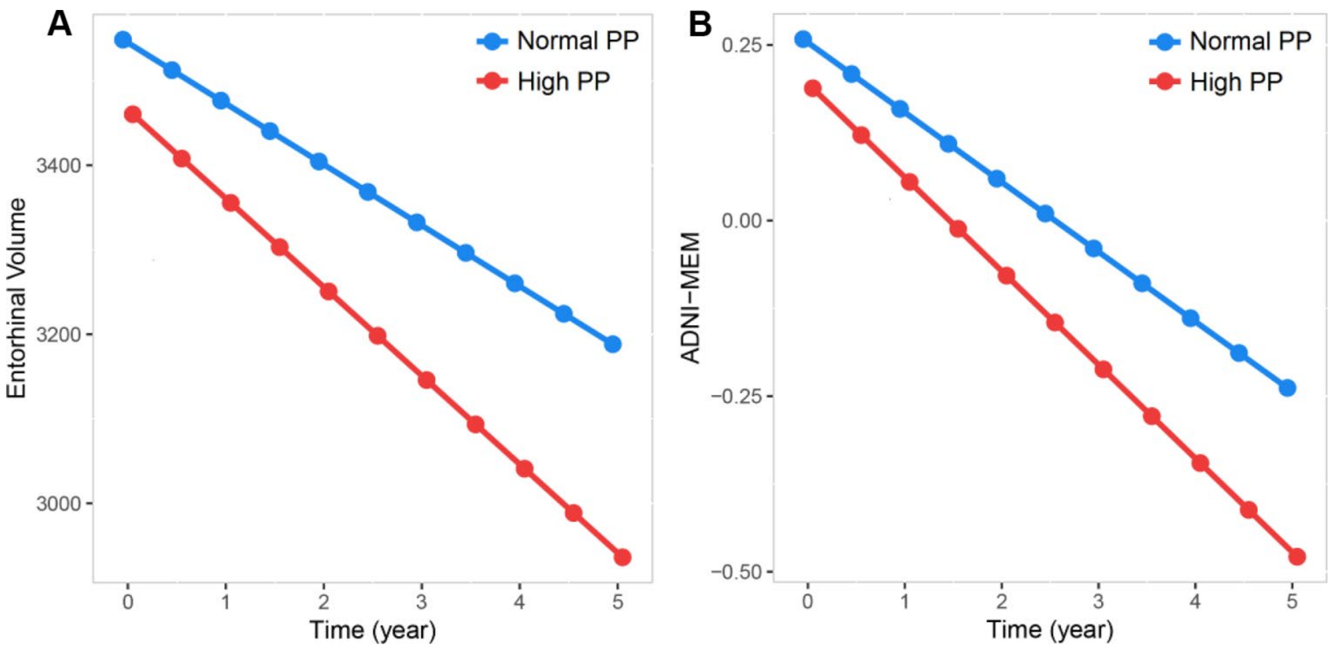

Supplementary Figure 1. Associations between baseline pulse pressure and brain aging measurements in cognitive diagnosis subgroups. Data from linear mixed-effects models adjusted for age, gender, education, *APOE* Ɛ4 carrier status, vascular risk factors, cognitive diagnosis, as well as intracranial volume. (**A**, **B**) Increased PP level was associated with an accelerated decline in entorhinal volume and episodic memory within mild cognitive impairment group.
