# Supplementary material for: High pulse pressure is a risk factor for prodromal Alzheimer’s disease: a longitudinal study
Source: Aging (Albany NY). 2020 Sep 22;12(18):18221–37. doi: 10.18632/aging.103678 (PMC7585106; doi:10.18632/aging.103678)
Supplement: Supplementary Tables [file aging-12-103678-s002..pdf]

SUPPLEMENTARY TABLES

Supplementary Table 1. Participant demographic and clinical information of CSF , AV45 PET imaging and MRI measurements analyses.

Participant features	CSF biomarkers analyses			AV45 PET imaging analyses			MRI measurements analyses		
	Normal PP	High PP	*P* Value	Normal PP	High PP	*P* Value	Normal PP	High PP	*P* Value
**N**	517	460		381	358		583	554	
**Age(Mean ± SD, year)**	71.79±6.895	74.18±6.98	**<0.0001**	70.67±6.595	73.63±7.072	**<0.0001**	71.92±6.632	74.3±6.776	**<0.0001**
**Gender (M/F)**	287/230	250/210	0.715	201/179	180/178	0.457	325/258	296/258	0.433
**Education (Mean ± SD, year)**	16.2±2.723	16.17±2.679	0.757	16.5±2.545	16.22±2.662	0.164	16.21±2.798	15.92±2.837	0.068
***APOE* Ɛ4 carrier status (2/1/0)**	301/174/42	273/156/31	0.711	220/131/30	215/119/24	0.746	331/201/51	328/190/36	0.338
**BMI (Mean ± SD, kg/m^2)**	27.22±4.95	27.16±4.751	0.827	27.84±5.175	27.74±5.168	0.636	27.08±4.732	27.06±4.82	0.697
**CVD(yes/no)**	103/416	89/371	0.845	50/331	55/303	0.384	129/454	124/430	0.918
**Hyperlipemia (yes/no)**	246/271	226/214	0.243	190/191	177/181	0.908	272/311	262/292	0.830
**Hypertension (yes/no)**	211/306	233/227	**0.002**	156/225	183/175	**0.006**	245/338	281/273	**0.003**
**T2DM (yes/no)**	31/486	38/402	0.116	27/354	34/324	0.234	40/543	47/507	0.304
**Cognitive diagnoses**									
**CN/MCI**	192/326	173/287	0.830	140/241	140/218	0.509	223/360	220/334	0.614

Abbreviations: Normal PP=normal pulse pressure; High PP=high pulse pressure; CSF, cerebrospinal fluid; SD=standard deviation; *APOE*, *apolipoprotein epsilon*; BMI ,Body Mass Index; CVD, Cardiovascular Disease; T2DM, Type 2 diabetes mellitus; CN ,cognitively normal; MCI, mild cognitive impairment.

Supplementary Table 2. Association between PP and CSF biomarkers and AV45 PET imaging in cross-sectional and longitudinal analyses.

Variables	Total		cognitive diagnostic strata				Age subgroups			
			CN		MCI		Young old group		Very old group	
	β	*P* Value	β	*P* Value	β	*P* Value	β	*P* Value	β	*P* Value
**Cross-sectional outcomes**										
**Aβ_{42}**	-0.525	**0.015**	-0.477	0.923	-0.068	**0.002**	-0.483	0.084	-0.063	**0.019**
**T-tau**	0.077	**0.011**	0.064	0.154	0.104	**0.009**	0.035	0.084	0.102	**0.031**
**P-tau**	0.097	**0.003**	0.112	**0.026**	0.099	**0.018**	0.078	0.062	0.111	**0.024**
**T-tau/ Aβ_{42}**	0.131	**0.004**	0.094	0.309	0.155	**0.002**	0.141	**0.041**	0.156	**0.009**
**P-tau/Aβ_{42}**	0.157	**0.001**	0.230	**0.047**	0.159	**0.004**	0.142	0.065	0.201	**0.002**
**Summary SUVR**	0.018	**0.011**	-2.888*e^{-4}	0.974	0.034	**0.003**	0.027	**<0.001**	0.025	0.211
**Longitudinal outcomes**										
**Aβ_{42}**	0.079	0.919	0.075	0.960	-0.035	0.965	1.458	0.161	-1.650	0.138
**T-tau**	-1.032	0.183	-0.279	0.750	-1.485	0.191	-1.477	0.140	-0.028	0.980
**P-tau**	0.201	0.759	1.617	0.096	-0.833	0.365	-1.061	0.241	1.669	0.134
**T-tau/ Aβ_{42}**	-0.010	0.123	-4.239*e^{-3}	0.468	-0.015	0.119	-0.020	**0.019**	0.004	0.707
**P-tau/Aβ_{42}**	-7.114*e^{-4}	0.889	9.941*e^{-3}	0.123	-8.105*e^{-3}	0.273	-0.012	0.090	0.013	0.104
**Summary SUVR**	1.457*e^{-3}	0.144	3.277*e^{-4}	0.840	2.293*e^{-3}	0.069	1.525*e^{-3}	0.221	1.033*e^{-4}	0.953

Abbreviations: PP, pulse pressure; CSF, cerebrospinal fluid; CN ,cognitively normal; MCI, mild cognitive impairment; Young old group, <75 years old; Very old group, ≥75 years old; Aβ_{42}, β-amyloid 42; T-tau ,total tau; P-tau, phosphorylated tau.

Supplementary Table 3. Association between PP and brain aging biomarkers in cross-sectional and longitudinal analyses.

Variables	Total		cognitive diagnostic strata				Age subgroups			
			CN		MCI		Young old group		Very old group	
	β	*P* Value	β	*P* Value	β	*P* Value	β	*P* Value	β	*P* Value
**Cross-sectional outcomes**										
**Hippocampal Volume**	$-1.084*e^{3}$	0.312	$-1.242*e^{1}$	0.863	$-8.861*e^{1}$	0.222	$-1.114*e^{4}$	0.251	$-8.833*e^{1}$	0.279
**Entorhinal Volume**	$-2.311*e^{1}$	0.554	$-1.053*e^{1}$	0.848	$-3.245*e^{1}$	0.545	$-1.522*e^{1}$	0.750	$-3.575*e^{1}$	0.592
**Mid-temporal Volume**	$4.364*e^{1}$	0.752	$1.030*e^{2}$	0.606	$2.378*e^{1}$	0.900	$1.085*e^{2}$	0.558	$-9.282*e^{1}$	0.663
**ADNI-MEM**	-0.030	0.343	-0.016	0.729	-0.031	0.467	-0.092	**0.036**	0.036	0.437
**ADNI-EF**	-0.042	0.298	-0.074	0.235	-0.015	0.777	-0.090	0.103	-0.005	0.940
**Longitudinal outcomes**										
**Hippocampal Volume**	-16.903	**0.039**	-12.534	0.222	-18.935	0.094	-11.173	0.307	-19.181	0.121
**Entorhinal Volume**	-20.014	**0.031**	-2.800	0.837	-32.776	**0.007**	-23.673	**0.046**	-12.120	0.426
**Mid-temporal Volume**	-23.890	0.382	-39.120	0.167	-16.890	0.664	-54.620	0.128	17.348	0.683
**ADNI-MEM**	$-1.829*e^{-2}$	**0.058**	0.001	0.896	-0.034	**0.012**	-0.016	0.214	-0.009	0.526
**ADNI-EF**	$1.395*e^{-4}$	0.991	$4.115*e^{-5}$	0.998	$-6.049*e^{-4}$	0.972	-0.001	0.937	0.013	0.409

Abbreviations: PP, pulse pressure; CN, cognitively normal; MCI, mild cognitive impairment; Young old group, <75 years old; Very old group, ≥75 years old.

Supplementary Table 4. Association between SBP and AD biomarkers and brain aging in cross-sectional analyses.

Variables	Total		cognitive diagnostic strata				Age subgroups			
			CN		MCI		Young old group		Very old group	
	β	*P* Value	β	*P* Value	β	*P* Value	β	*P* Value	β	*P* Value
**$Aβ_{42}$**	-0.373	**0.014**	-5.575	0.113	-0.027	0.065	-0.164	0.417	-0.061	**<0.001**
**T-tau**	0.047	**0.026**	0.024	0.464	0.073	**0.008**	0.006	0.685	0.106	**0.001**
**P-tau**	0.060	**0.007**	0.077	**0.032**	0.060	**0.037**	0.050	0.097	0.082	**0.014**
**T-tau/ $Aβ_{42}$**	0.084	**0.008**	0.063	0.341	0.098	**0.006**	0.044	0.370	0.148	**<0.001**
**P-tau/$Aβ_{42}$**	0.106	**0.002**	0.223	**0.007**	0.081	**0.034**	0.078	0.160	0.151	**<0.001**
**Summary SUVR**	0.011	**0.037**	0.003	0.596	0.016	**0.041**	0.015	**0.003**	0.012	0.362
**Hippocampal Volume**	$-3.019*e^{2}$	0.690	$4.465*e^{1}$	0.392	$-6.319*e^{1}$	0.209	$-1.711*e^{3}$	0.809	$-2.560*e^{1}$	0.642
**Entorhinal Volume**	$-3.313*e^{1}$	0.228	$-2.351*e^{1}$	0.556	$-3.582*e^{1}$	0.334	$-4.856*e^{1}$	0.162	$-1.369*e^{1}$	0.761
**Mid-temporal Volume**	$-1.837*e^{2}$	0.059	$6.416*e^{1}$	0.658	$-3.200*e^{2}$	**0.014**	$-2.062*e^{2}$	0.127	$-1.379*e^{2}$	0.337
**ADNI-MEM**	-0.036	0.108	-0.029	0.386	-0.040	0.175	-0.064	**0.042**	$-4.794*e^{-4}$	0.988
**ADNI-EF**	-0.055	0.053	-0.036	0.424	-0.063	0.079	-0.080	**0.046**	-0.035	0.396

Abbreviations: SBP, systolic blood pressure; CN ,cognitively normal; MCI, mild cognitive impairment; Young old group, <75 years old; Very old group, ≥75 years old; $Aβ_{42}$, β-amyloid 42; T-tau ,total tau; P-tau, phosphorylated tau.

Supplementary Table 5. Association between DBP and AD biomarkers and brain aging in cross-sectional analyses.

Variables	Total		cognitive diagnostic strata				Age subgroups			
			CN		MCI		Young old group		Very old group	
	β	*P* Value	β	*P* Value	β	*P* Value	β	*P* Value	β	*P* Value
**$A\beta_{42}$**	0.018	0.923	-3.756	0.394	0.017	0.346	0.217	0.369	-0.019	0.426
**T-tau**	-0.016	0.537	0.055	0.174	-0.063	0.063	-0.016	0.361	-0.021	0.609
**P-tau**	-0.033	0.232	-0.009	0.832	-0.036	0.307	-0.039	0.279	-0.014	0.755
**T-tau/ $A\beta_{42}$**	-0.017	0.662	0.126	0.136	-0.068	0.114	-0.059	0.316	-0.002	0.969
**P-tau/$A\beta_{42}$**	-0.033	0.437	0.027	0.793	-0.048	0.300	-0.070	0.291	-0.005	0.933
**Summary SUVR**	-0.005	0.460	0.006	0.425	-0.001	0.246	-0.005	0.425	0.003	0.835
**Hippocampal Volume**	$1.447*e^{3}$	0.109	$-7.129*e^{1}$	0.247	$-6.045*e^{1}$	0.316	$3.431*e^{2}$	0.967	$-5.790*e^{1}$	0.396
**Entorhinal Volume**	$-3.808*e^{1}$	0.246	$-5.520*e^{1}$	0.242	$-1.974*e^{1}$	0.657	-1.814	0.964	$-6.853*e^{1}$	0.219
**Mid-temporal Volume**	$-3.337*e^{2}$	**0.004**	$-3.049*e^{2}$	0.074	$-3.178*e^{2}$	**0.042**	$-2.807*e^{2}$	0.072	$-2.895*e^{2}$	0.104
**ADNI-MEM**	-0.055	**0.044**	-0.067	0.091	-0.040	0.277	-0.015	0.698	-0.084	**0.034**
**ADNI-EF**	-0.055	0.117	0.026	0.625	-0.099	**0.028**	-0.024	0.621	-0.075	0.145

Abbreviations: DBP, diastolic blood pressure; CN ,cognitively normal; MCI, mild cognitive impairment; Young old group, <75 years old; Very old group, ≥75 years old; $A\beta_{42}$, β-amyloid 42; T-tau ,total tau; P-tau, phosphorylated tau.

Supplementary Table 6. Association between Hypertension and AD biomarkers and brain aging in cross-sectional analyses.

Variables	Total		cognitive diagnostic strata				Age subgroups			
			CN		MCI		Young old group		Very old group	
	β	*P* Value	β	*P* Value	β	*P* Value	β	*P* Value	β	*P* Value
**$A\beta_{42}$**	-1.916	0.529	3.783	0.456	-0.023	0.301	-0.320	0.273	-0.011	0.687
**T-tau**	-3.578	0.169	-0.054	0.239	-0.012	0.764	-0.026	0.217	0.007	0.889
**P-tau**	-1.104	0.359	-0.023	0.652	-0.012	0.782	-	-	-	-
**T-tau/ $A\beta_{42}$**	-0.032	0.498	-0.109	0.255	0.013	0.801	-0.029	0.682	0.017	0.778
**P-tau/$A\beta_{42}$**	-0.009	0.857	-0.079	0.512	0.020	0.727	0.040	0.615	-0.021	0.751
**Summary SUVR**	-0.005	0.531	-0.006	0.538	-0.002	0.841	0.008	0.290	-0.023	0.277
**Hippocampal Volume**	$-5.023*e^{3}$	0.186	$-8.436*e^{1}$	0.313	$-7.653*e^{1}$	0.400	$-2.357*e^{2}$	**0.008**	$2.108*e^{5}$	0.753
**Entorhinal Volume**	$-5.063*e^{1}$	0.269	$-8.490*e^{1}$	0.182	$-1.631*e^{1}$	0.799	$-1.221*e^{2}$	**0.033**	$2.820*e^{1}$	0.714
**Mid-temporal Volume**	$-2.049*e^{2}$	0.213	$-9.223*e^{1}$	0.698	$-2.500*e^{2}$	0.264	$-5.001*e^{2}$	**0.021**	$1.158*e^{2}$	0.658
**ADNI-MEM**	-0.063	0.103	-0.063	0.247	-0.069	0.190	-0.125	**0.018**	-0.032	0.570
**ADNI-EF**	-0.163	**0.001**	-0.122	0.112	-0.182	**0.005**	-0.245	**<0.001**	-0.159	**0.031**

Abbreviations: CN ,cognitively normal; MCI, mild cognitive impairment; Young old group, <75 years old; Very old group, ≥75 years old; $A\beta_{42}$, β-amyloid 42; T-tau ,total tau; P-tau, phosphorylated tau.
